# Cross-sectional study of hepatitis E virus (HEV) circulation in Italian pig farms

**DOI:** 10.3389/fvets.2023.1136225

**Published:** 2023-04-18

**Authors:** Giovanni Ianiro, Enrico Pavoni, Giuseppe Aprea, Romina Romantini, Giovanni Loris Alborali, Daniela D'Angelantonio, Giuliano Garofolo, Silvia Scattolini, Luca De Sabato, Chiara Francesca Magistrali, Elke Burow, Fabio Ostanello, Richard Piers Smith, Ilaria Di Bartolo

**Affiliations:** ^1^Department of Food Safety, Nutrition and Veterinary Public Health, Istituto Superiore di Sanità, Rome, Italy; ^2^Istituto Zooprofilattico Sperimentale della Lombardia e dell'Emilia-Romagna “Bruno Ubertini”, Brescia, Italy; ^3^Istituto Zooprofilattico Sperimentale dell'Abruzzo e del Molise “Giuseppe Caporale”, Teramo, Italy; ^4^Istituto Zooprofilattico Sperimentale dell'Umbria e delle Marche “Togo Rosati”, Perugia, Italy; ^5^Department of Biological Safety, German Federal Institute for Risk Assessment, Berlin, Germany; ^6^Department of Veterinary Medical Sciences, University of Bologna, Ozzano dell'Emilia, Italy; ^7^Department of Epidemiological Sciences, Animal and Plant Health Agency, Weybridge, United Kingdom

**Keywords:** hepatitis E virus, HEV, zoonoses, pig, feces, farm, fattening, surveillance

## Abstract

Foodborne transmission is considered the main way of spreading zoonotic hepatitis E virus (HEV) infection in Europe. In recent years, the human cases of hepatitis E in subjects without history of travel in endemic areas have raised, suggesting that domestic HEV transmission is increasing. Pork products with or without liver, are often indicated as the source of many human foodborne HEV cases as well as small outbreaks. Pigs are recognized as the main reservoir of the zoonotic HEV-3 genotype, the most frequently detected in human cases in the EU. In the absence of a harmonized surveillance of HEV circulation, data on prevalence are heterogeneous but confirm a widespread circulation of HEV-3 in pig herds across EU. HEV-3 can pass through the food chain from farm to fork when infected animals are slaughtered. In Italy, several studies reported the circulation of HEV-3 in pig farms, but results are heterogeneous due to different methodologies applied. In the present study, we performed a survey over 51 pig herds belonging to three main types of farms: breeding, fattening and farrow-to-finish. HEV-RNA was analyzed by broad range Real-time RT-PCR on 20 samples for each farm, obtained by pooling together feces from 10 individuals. Overall, HEV RNA was confirmed on 150 fecal pooled samples out of 1,032 (14.5%). At least one positive pooled sample was detected from 18 farms out of 51 tested (35.3%). By lowering the number of infected pigs at primary production, the risk of HEV-3 entering into the food chain can be reduced. Hence, information on HEV circulation in herds is highly relevant for choosing preventive measures and deserves development of a monitoring program and further investigations.

## Introduction

In recent years, an increasing number of autochthonous human cases of hepatitis E have been reported in Europe (1). The disease is generally self-limiting but can become chronic in fragile groups, such as immunocompromised or organ transplant patients. The disease was considered for a long time only associated with travel to endemic areas, where large waterborne outbreaks often occur (2). Only recently, due to an increase of cases in the EU, it has become clear that autochthonous cases also occur (2). In the EU, most cases are associated with the consumption of raw or undercooked meat of animal origin, mainly containing liver of pork or wild boar (1). The disease has a different epidemiology in low-income countries, causing large outbreaks, without chronic sequalae of the disease, but with a high mortality rate in pregnant women (3). In developed countries, sporadic cases or small clusters have been mainly described, and chronic hepatitis can occur (4).

The aetiological agent is the hepatitis E virus (HEV), a single stranded RNA virus. The HEV genotypes HEV-1 and HEV-2 only infect humans and circulate in low-income countries, whereas genotypes HEV-3 and HEV-4 infect both humans and animals and circulate worldwide. In the EU, genotype HEV-3 is the most common in human cases and in animals, with pigs and wild boar as main reservoir (5). Pigs can be also infected by HEV-4 which circulates mainly in Asia, and has been sporadically retrieved in pigs in Italy (6) and Belgium (7, 8). In pigs and wild boar, the infection does not cause any symptoms, making the identification of HEV positive animals difficult.

The zoonotic transmission of HEV is now recognized and supported by experimental infections that have proved that the swine HEV-3 strain can successful infect non-human primates (9), while foodborne transmission is supported by epidemiological evidence and identification of the same strains in human cases and leftover food (9, 10). In recent years in the EU, the number of autochthonous confirmed human cases increased more than 3-fold between 2005 and 2015 (1). The reason could be the increased awareness of clinicians or better diagnostic tests, but may also be linked to the spreading of HEV in animal reservoirs and the raising of novel variants, called subtypes, better adapted to humans.

HEV-3 is present worldwide in pigs, the infection can be spread via direct contact with another infected pig or contaminated feces. The estimated basic reproduction number is very high (*R*_0_ = 8.8) determining a large spreading of the virus (11). Pigs are susceptible to the infection from the age of 2 months, when the maternal immunity decreases. After infection, the virus replicates in the liver, a transient viremia with IgM response is described, together with fecal shedding of the virus. The infection induces production of IgG which can reach a peak of 100% in adult animals within the same farm (12). However, it is unclear if the immunity is protective and if animals can be re-infected during their life, since some studies reported the presence of the virus in finishers at farms or at the stage of slaughter in feces (13, 14), liver (15, 16) and, only in small number of cases, in blood (17, 18). The presence of HEV in muscle is rare and probably linked to a stage of viremia and insufficient bleeding at slaughter (13, 16). The main risk for consumers is linked to consumption of raw or uncooked pork, mainly containing liver (1). Some cases in the EU were linked to the consumption of liver sausages (*figatellu*) (1) and wild boar meat (19).

The prevalence at farm level ranges between 10.0 and 100% and at individual level from 1.0 to 89.0% in the 69 studies conducted over the world (12). In Italy, the first report of HEV-3 in pigs was published in 2005 (20), followed by several studies reporting variable individual prevalence in pig feces at farms. HEV-RNA was detected in 41.9% of 274 randomly selected pigs from six different swine farms in Northern Italy raising from 12.5 to 72.5% (21). A similar result with individual prevalence of 41.5% was reported in 2015 in a study conducted on 17 farms in the same geographical area (22). The farm prevalence was also variable, from 24.8% (26 positive farms out of 105 tested) (23) to 100%, when only 6 farms were investigated (21).

This variability of prevalence reported in Italy and elsewhere could be linked to differences in methodologies and sampling (age of animals or population investigated) which could largely influence the prevalence observed (12).

The present study aimed to define the current HEV prevalence in Italian pig farms. To this purpose, we investigated the occurrence of HEV in 51 pig farms located either in Northern Italy, where large intensive pig farms are common, or from Central Italy, where smaller pig farms are present. Breeding, fattening as well as farrow-to-finish farms, were included. The scope of the study was to determine the occurrence of HEV in pigs in Italy by determining the within farm prevalence and the difference in the occurrence of HEV among animal category (breeding *vs* fatteners). As a secondary aim, differences related to the type of farms and their size were also assessed. A common methodology of sampling and analyses was conducted throughout the study to have a reliable farm prevalence across the country.

## Materials and methods

### Farms enrolment

**Figure 1:**
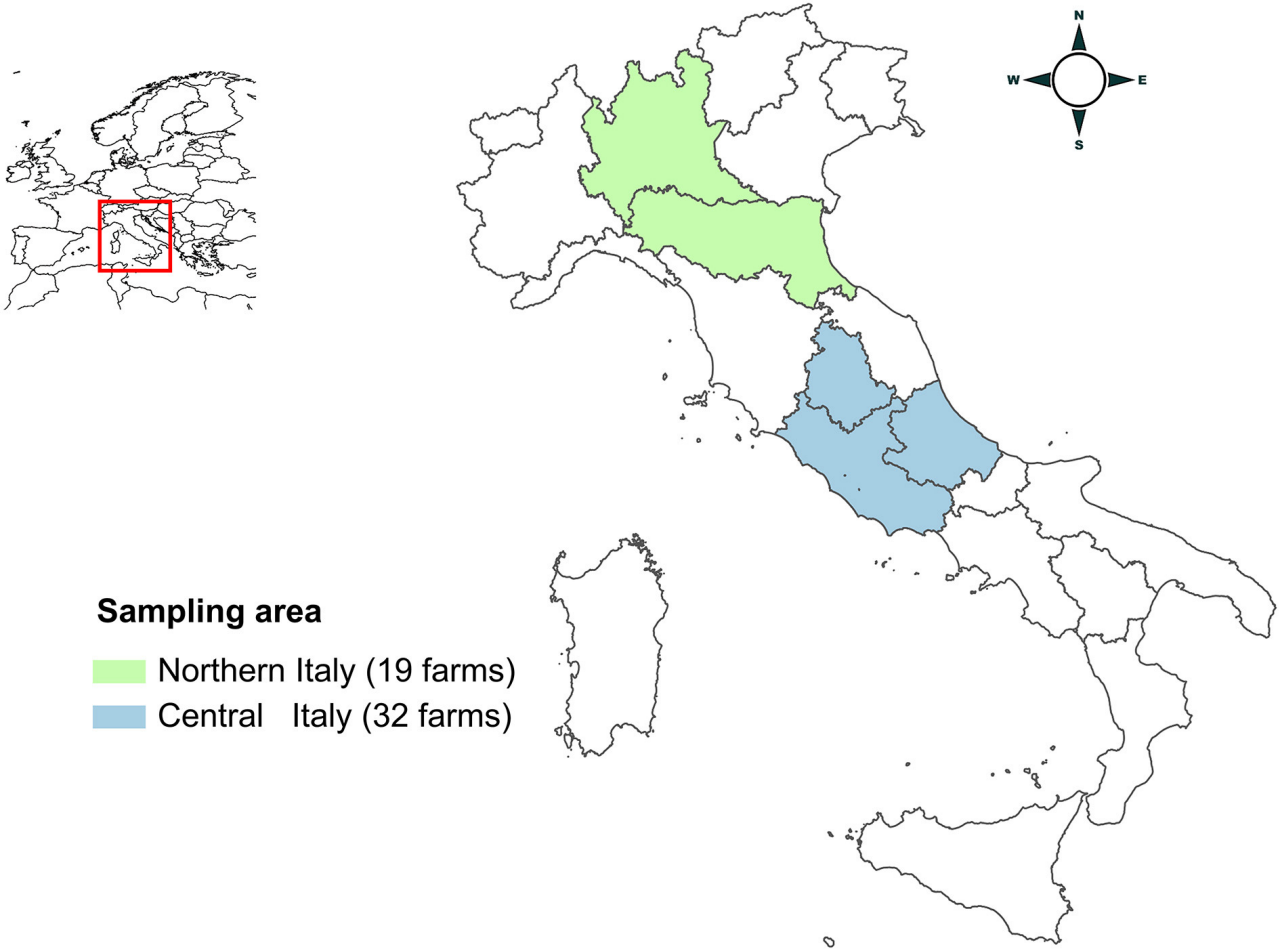
Map of Italy showing the areas of sampling. Lombardia and Emilia-Romagna regions (Northern Italy) are highlighted in green; Lazio, Umbria and Abruzzo regions (Central Italy) are highlighted in blue.

Three laboratories (Istituto Superiore di Sanità, ISS; Istituto Zooprofilattico Sperimentale dell'Abruzzo e del Molise, IZSAM; Istituto Zooprofilattico Sperimentale della Lombardia e dell'Emilia-Romagna, IZSLER) participated in the study, receiving field samples for processing. During 2019-2021, 51 Italian conventional pig farms (26 fattening, 18 breeding and 7 farrow-to-finish) were enrolled in the study without regard to previous history of HEV infection. Convenience sampling was performed, based upon relevant farms being present in the regions of Northern and Central Italy and their willingness to participate. Farms were located in Northern (11 Lombardia and 8 Emilia-Romagna Regions) and Central Italy (26 Abruzzo, 4 Lazio, and 2 Umbria Regions) (Figure 1). Nucleus/multiplier herds producing replacement breeding pigs for breeding farms or specific pathogen free (SPF) farms, were not sampled, due to their limited numbers and expected difficulties in ability to access these farms. Based on the median number of sows or reared animals, farms were grouped as “small” or “large.” Breeding farms and farrow-to-finish farms were considered large if the number of sows was >1,750 and >200, respectively. Fattening farms were considered large when the number of animals present was >700.

### Sampling scheme

For each farm (*n* = 51), 20 fecal samples were obtained by pooling together feces from 10 individuals, except for three farms where 24 samples were collected, resulting in 1,032 pooled samples. Samples were taken individually from fresh feces, preferably immediately after defecation, on the pen floor, with each single sample containing a minimum of 10 g of feces. The whole pooled sample was collected in a sterile plastic bag with specific disposable plastic spoons or pliers. Gloves and sampling material were changed between each pooled sample collection. For each pooled sample, feces were collected to represent an area or a pig age group present on the farm. The pooled samples were collected to represent as many pens as possible into the farm and these were joined together before transport and stored at −20°C until use.

Analyses of 20 pooled samples were planned to have a sensitivity to detect at least one positive sample with a within-herd prevalence of ≥2% (assuming a test sensitivity of 90% for all pooled sample sizes and to provide a desired cluster-sensitivity of 95%). The same number of pooled samples (20, with 10 individual samples) can be used to estimate HEV prevalence assuming a perfect test (100% sensitivity and specificity) an estimated true prevalence of 10%, a desired precision (allowable error) of 5.5% and 95% of C.I. (24).

The number and type of fecal samples to be examined were established in the framework of the One Health European Joint Programme, BIOPIGEE project, and shared with the other project participant's partners (9 countries). The decision was the result of consultation of HEV and *Salmonella* experts, since the samples were also analyzed for *Salmonella*. On fattening farms (*n* = 26), 20 pooled samples were collected from finishers, at an age of 5-7 months, which represents an age close to the slaughter of pigs, which in Italy is usually 9 months. On breeding farms (*n* = 18), 16 samples from gilts and four from dry sows were collected. On farrow-to-finish farms (*n* = 7), 10 pooled samples from finishers (5–7 months), eight from gilts and two from dry sows were collected.

Even when the number of pigs present was less than 200 (3 farms located in Central Italy), the same sampling scheme was followed.

### RNA extraction from fecal pooled samples

One hundred mg of homogenized fecal samples were suspended by vortexing in RNAase free water to the final 10% (w/v) ratio. After centrifugation at 5.000 × g for 10 min, fecal supernatants were recovered, of which 100 μL were used for RNA extraction. Two laboratories used the NucliSens MiniMAG platform with the NucliSens magnetic extraction Kit (BioMerieux, Marcy-l'Étoile, France) and one laboratory used QiaCube automated system with the Qiamp Viral Mini kit (Qiagen, Milan Italy) according to the manufacturer's instruction, except for the final volume of elution that was set to 100 μL in all laboratories. Before RNA extractions, supernatants were spiked with Mengovirus (process control virus, provided by the National Reference Laboratory for Foodborne Viruses, Istituto Superiore di Sanità, Rome, Italy) or Murine Norovirus (MuNoV strain IT-1, Istituto Superiore di Sanità, Rome, Italy), as previously described (13, 25).

### Nucleic acid recovery rate calculation

The RNA extraction recovery rate (RR) was estimated by the comparative cycle threshold (Ct) method using the Ct values of the RNA of Mengovirus or MuNoV used to artificially spike samples (26). RNA extractions were considered acceptable with a RR ≥1%.

### Real-time reverse transcription PCR for HEV detection

For HEV detection, 5 μL of total RNA were analyzed using the RNA UltraSense™ One-Step qRT-PCR System (Thermofisher Scientific, Frederick, MD, USA), as previously described (13, 27), with primers HEV-F (5'-GGTGGTTTCTGGGGTGAC-3'), HEV-R (5'-AGGGGTTGGTTGGATGAA-3') and HEV probe (TaqMan HEV-R probe, 5' -FAM-TGATTCTCAGCCCTTCGC-BGQ1-3').

### Limit of detection (LOD) estimation of HEV-RNA detection methods

Serial 2-fold dilutions of 1st WHO International Standard for Hepatitis E Virus (genotype 3a; PEI code 6329/10; Paul-Ehrlich Institute, Germany) from 12,500 IU to 390 IU (corresponding to 4 dilutions from 70,100 genome equivalents, GE, to 1,095 GE; 5.39 log_10_ copies/mL) (28) were prepared to spike in triplicate fecal supernatants, previously tested as HEV-negative. From the spiked samples, 100 μL of supernatants, were subjected to RNA extractions as described above. The LOD_100_ was estimated as the lowest dilution detectable by the methods used in all the three replicates by testing 5 μL of RNA (350.68 GE to 5.48 GE).

### Statistical analysis

The difference in the proportion of HEV positive farms (positive for HEV if at least one pooled sample tested positive) was statistically analyzed. The analyses were conducted by size (farms were categorized as small or large depending on the number of pigs or sows present) and by geographic localization (North or Central Italy) within each farm typology, using the Pearson Chi-Square test. For farms with at least one pooled sample positive for HEV, the within farm prevalence and 95% exact confidence limits were estimated from the pooled samples following binomial theory (24) and employing a pooled prevalence calculator (Epitools Epidemiological Calculators. Ausvet. Available at: https://epitools.ausvet.com.au).

The Kolmogorov-Smirnov test (K-S) for goodness of fit was used to verify normality of the estimated prevalence (proportion of positive samples) data distribution. After confirming a not-normal distribution, the Mann-Whitney U-test was used to compare the estimated prevalence values between farm typology, and dimension.

Statistical significance was set at *p* ≤ 0.05. All statistical analyses were performed using the software SPSS 28.0.0 (IBM SPSS Statistics, Armonk, NY, USA).

## Results

Before starting the survey, in the three involved laboratories, the LOD_100_ was estimated as 5.48 copies of HEV corresponding to 1.10 × 10^4^ GE/g in feces. Furthermore, the recovery rate of extractions was estimated for the 437 valid extractions using either Mengovirus or MuNoV as the process control, spiked in the pooled fecal samples. Samples with >1% recovery rate were used for the detection of HEV. The mean recovery rate was 65.5%. The mean recovery rate obtained was 52.6 % for mengovirus extracted with the Nuclisens kit, and 76.6 % for murine norovirus extracted with the Qiagen kit.

Eighteen out of the 51 farms (35.3%) had at least one pooled sample positive for HEV. The fattening farms were the most frequently positive for HEV (11/26, 42.3%), followed by farrow-to-finish (2/7, 28.6%) and breeding farms (5/18, 27.8%) (Table 1).

**Table 1:** HEV positive farms grouped by type, size and geographic localization.

**Farm type**	**Dimension**	**Total tested farms**	**Number of HEV positive farms (%)^a^**	***p***
Breeding	Small (<1,750 sows)	9	3 (33.3)	1.00
	Large (≥1,750 sows)	9	2 (22.2)	
	Total	18	5 (27.8)	
Farrow-to-Finish	Small (<200 sows)	3	1 (33.3)	1.00
	Large (≥200 sows)	4	1 (25.0)	
	Total	7	2 (28.6)	
Fattening	Small (<700 pigs)	14	7 (50.0)	0.45
	Large (≥700 pigs)	12	4 (33.3)	
	Total	26	11 (42.3)	
Total	Small	26	11 (42.3)	
	Large	25	7 (28.0)	0.28
	Total	51	18 (35.3)	
Breeding	Northern Italy^b^	12	3 (25.0)	1.00
	Central Italy^c^	6	2 (33.3)	
	Total	18	5 (27.8)	
Farrow-to-Finish	Northern Italy	1	0 (0.0)	1.00
	Central Italy	6	2 (33.3)	
	Total	7	2 (28.6)	
Fattening	Northern Italy	4	3 (75.0)	0.28
	Central Italy	22	8 (36.4)	
	Total	26	11 (42.3)	
Total	Northern Italy	17	6 (35.3)	1.00
	Central Italy	34	12 (35.3)	
	Total	51	18 (35.3)	

a At least one HEV positive sample;

b Lombardia, Emilia-Romagna Regions;

c Abruzzo, Lazio, Umbria Regions.

However, by analyzing the proportion of positive farms, there was no significant differences observed for HEV occurrence and farm size when all the farms were grouped together (Table 1) (Pearson Chi-Square = 1.14; *p* > 0.28) or when farms were grouped by type (breeding: Pearson Chi-Square = 0.28, *p* = 1; farrow-to-finish: Pearson Chi-Square = 0.06, *p* = 1.00; fattening: Pearson Chi-Square = 0.73 *p* = 0.45).

**Figure 2:**
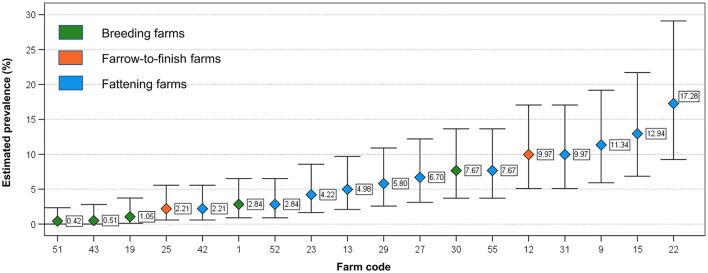
Distribution of estimated prevalence in the examined HEV positive herds (with at least 1 positive pooled sample). Whiskers indicate 95% Confidence Interval.

Similarly, no significant differences were observed between HEV occurrence and the geographical location of the farms (Pearson Chi-Square = 0.00; *p* = 1.00) grouped together or when farm types were group by type, (*p* > 0.05) (Table 1). The proportion of positive pooled samples were used for calculating the estimated prevalence, which widely varied within fattening, farrow-to-finish and breeding farms (Figure 2). The mean values were of 7.0% (95% Confidence Interval, 95%CI: 5.7–8.3), 5.4 % (95% CI: 3.1–8.5) and 2.1% (95% CI: 1.3–3.2), respectively (Figure 2). The only significant difference in estimated farm prevalence was detected for farm type, which was greater in fattener farms compared to the other farm types (Mann-Whitney U = 16.00*;* −0.04).

Overall, evidence of HEV RNA was confirmed on 150 fecal pooled samples out of 1,032 (14.5%). Percentages of pooled samples positive for HEV within farms varied from 20.0% (4 out of 20) to 85.0% (17 out 20) for the 11 positive fattening farms (median of positive pooled samples, 10) (Table 2). All 11 fattening farms had ≥20% positive pooled samples. In the 5 HEV positive breeding farms, the proportion of positive pooled samples ranged from 4.2 % (1 out of 24) to 55.0% (11 out of 20) (median 2), and for the two farrow-to-finish HEV positive farms 20.0% (4 out of 20) and 65.0% (13 out of 20) positive pooled samples were detected, respectively (Table 2).

**Table 2:** Summary of results obtained for HEV-detection for each farm in the pooled samples of feces.

**Type of farm**	**Number of HEV positive farms/total**	**Total pooled samples tested**	**Number of HEV positive pooled samples (%)**	**Min–max number of positive pooled samples/farm**	**Median of positive pooled samples per farm**
Fattening	11/26	520	113 (21.7)	4–17	10
Breeding	5/18	372^a^	20 (5.4)	1–11	2
Farrow to finish	2/7	140	17 (12.1)	4–13^b^	8.50
Total	18/51	1032	150 (14.5)	1–17	8.50

a for three breeding farms (3/23) 24 pooled samples were collected rather than 20;

b only two farms positive.

Considering the type of pigs sampled, HEV positivity was higher in fattener pigs (pooled samples of fattener pigs from finisher and farrow to finish farms, 120/588 pool samples 20.4%) than in breeding pigs (pooled samples from dry sows and gilts on farrow-to-finish and breeding farms, 30/444; 6.7%; Pearson Chi-Square = 37.95; *p* < 0.001).

## Discussion

In the EU, the main route of HEV-3 zoonotic transmission is foodborne. Pork products with and without liver are the most frequently associated sources of HEV cases in the EU, if consumed raw or undercooked (10). In Italy, national studies on anti-HEV IgG positivity in blood donors, reported a mean prevalence of 8.3% (29). A risk factor associated with the consumption of raw pork sausages with liver has been evidenced, and the consumption of this product is common in some regions in Central Italy (29). The role of pork with liver as the source of infections was also reported in Italy (30, 31). In the Italian area with the highest density of pig farming (Northern), the mean seroprevalence in blood donors is in line with the rest of the country. Conversely, Abruzzo and Sardinia Regions are two hyper-endemic areas showing high seroprevalence in humans (27% and >10%, respectively) (29), but where only smaller pig herds are present (32). As already mentioned, the high seroprevalence in Abruzzo Region could be due to the habits to consume raw pork meat-and-liver sausages.

In the present study, using common methodologies for sampling and a common approach for HEV detection, 51 farms located in Northern and Central Italy were investigated to explore the distribution of HEV and generate information that can be used in the future for control strategies for HEV occurrence at farms. A pooled sample approach to estimate the presence of HEV in pigs was used in this study. The method used was assayed and showed a LOD_100_ of 5.48 GE/mL, corresponding to 1.1 × 10^4^ GE/g of pool of feces used for the analyses. In a previous study, we observed a sensitivity of sampling ranging between 2.0 × 10^2^ and 1.6 × 10^4^ GE/g, by pooling together 1 positive and 19 negative animals (33). This approach allowed for efficiencies of money and time and showed that, even if negatives can dilute the positive individual samples, the pooled fecal samples were still found to be sensitive for HEV detection. This demonstrated that the method was highly efficient (33).

Overall, in the present study, 14.5% of pooled fecal samples were HEV-positive and 18 out of 51 farms (35.3%) were positive for at least one pooled sample. From all farms, 20 pooled fecal samples were collected, including the 3 farms with less than 200 pigs. This could determine a bias of over-estimation of positivity in the three farms, albeit only one was HEV positive. The comparison between the two areas (Northern and Central Italy) was hampered by the different number of farms investigated within each area. However, the percentage of positive farms in Northern and Central Italy was identical, with 6/17, 35.3% and 12/34, 35.3%, respectively. Several previous studies conducted in Northern Italy reported a similar percentage of HEV positive farms. The percentage varied from 24.8%, 26 out of 105 farms tested (23) to 31.0% in 42 tested farms (34); higher values were observed in a group of 17 farms (52.9%) (22) and in a study involving 6 farms with all farms HEV positive (21). Conversely, farms in Southern Italy have been rarely investigated: studies were conducted on two, eight and twelve farms with a percentage of positive farms of 50.0%, 12.5% and 33.3%, respectively (35–37). Nevertheless, seroprevalence studies conducted on the same farms frequently showed 56.8% and up to 80.0% positivity in pigs, confirming a wide circulation of the virus also in farms in Southern Italy (35, 37). Among all these previous studies, two involved pooled fecal sampling, as it was used in the present study (22, 23).

Differences in pig production between Northern and Central-Southern Italy are significant. In Northern Regions, mainly in Lombardia and Emilia-Romagna, there is a higher number of pig herds, housing, respectively 46.6% and 16.2% of total pigs raised in Italy, in comparison to 2.3%, 0.9% and 0.5% in Umbria, Abruzzo and Lazio, respectively (Central Italy https://www.vetinfo.it/j6_statistiche/#/ accessed on 31/12/2022) (38). In the North, there are mainly intensive pig farms, with the main part of production allocated to export as raw hams or other long-cured products, whereas in the Central-South of Italy the pig herds host a smaller number of animals, with the main production being for local distribution (39). However, in this study, no significant difference was observed in the occurrence of HEV by comparing the farms for their size, within each type of farm. In the Abruzzo region, where 26 analyzed farms were located, small sized herds are present mainly for the Italian or local market. Abruzzo is an HEV genotype 3 hyperendemic area for humans (29) but this does not correspond either to a higher percentage of positive HEV herds (in this study) or to higher percentage of positive animals at slaughterhouse, as observed in a recent study conducted by HEV detection in liver on pigs at slaughter (40). The link between high seroprevalence in humans in Abruzzo and the consumption of raw pork liver (e.g., sausages) has been confirmed by epidemiological data (29). In a recent study, focused on an unexpected increase of number of HEV human cases in Abruzzo in 2019, the virological and the epidemiological investigation concluded that cases could have been caused by HEV strains newly introduced in the area, since the strains involved were never identified before in the area in human cases (41). This result confirms that the movement of pigs and offal makes tracing the sources of human cases very difficult.

In our study, fattening farms represent the farm type most frequently positive and the category of fattener pig was identified as the most likely to be positive. It is noteworthy that the estimated prevalence showed a marked variability within this category of farms, from 2.21% to 17.3 % in fattening farms (Figure 2). This variability was observed frequently in other studies. In 69 studies conducted worldwide it is reported an individual prevalence ranging from 1.0 to 89.0% (12). The observed differences in the prevalence could be linked to the different infection characteristics within farms influenced by several risk factors such as demographic characteristic, internal and external biosecurity measures (42), which needs to be investigated to mitigate the risk of occurrence of HEV at farm. In Italy, the within-farm prevalence in fattening farms varied between 20.0 to 62.5% (22) and from 11.1 to 100% (37). If results of the studies are compared, this variability could be linked to the methods used for the analyses, which are heterogenous, to a low number of animals tested per farm, but also to the age of pigs. In fact, pigs are generally moved to the fattening farms after weaning at the age of 11–13 weeks (25–35 kg live body weight), when the probability to be HEV-infected is higher, and spend about 6 months in the same farm before being slaughtered (from 5 to 9 months of age).

In this study, pooled fecal samples from finisher pigs were more likely to be positive than those from breeding pigs. In previous studies, seroprevalence higher than 25.0% of fattening pigs was considered a risk factor that could also determine a higher probability of viral presence in the liver at slaughtering (OR 6.7) (12, 43). Conclusions on this hypothesis are difficult since too many factors can influence the occurrence of infected animals at slaughter, but a higher presence of HEV in fattening farms deserves constant monitoring. In the study, finishers at 5–7 months of age (slaughter age) were tested, because of the interest in determining if finishers were at risk of HEV infection and may contaminate the food chain.

The reduction of HEV occurrence on primary production may be the most effective step to control the spreading of the infection. The data obtained in this study could be significant for the Competent Authorities and farmers to prevent HEV circulation, which could be achieved by applying improved biosecurity measures on farm level (44). Furthermore, this information could be used to enhance the knowledge on foodborne viral risk among consumers and human medicine. To achieve a plan of control, a wide monitoring of HEV occurrence is needed. This study is a first step toward this aim, but additional data are required.

The limits of this study were the non-homogenous farm enrolment over the whole country, and the different number of farms sampled for each category. These limits were partly due to the difficult access to farms for the concomitance of this study with the COVID-19 pandemic. Furthermore, since HEV surveillance is not mandatory, only farmers willing to participate contributed to the study. Concerning breeder pigs, a higher number of samples could have been collected, but the same number of 20 pooled samples were collected per farm, and number of pooled samples collected per pig type were uniform. This choice was made because samples were being investigated also for the presence of *Salmonella*, so this sampling was a compromise for the detection of both pathogens. The within farm prevalence was calculated by using the results of HEV investigation on pooled samples. The used approach could be considered controversial, but is supported by previous papers (45, 46) and is very useful when many farms are investigated. Future studies will be performed to assess the HEV genotypes circulating in the investigated areas of sampling.

## Conclusions

In conclusion, this study confirms that the hepatitis E virus (HEV) circulates in Italian pig farms but besides the high seroprevalence observed in previous studies (35, 37, 47), the detection of HEV-RNA, and proof of viral replication, is less frequent. Nevertheless, in this study the absence of correlation between occurrence of HEV at farm and neither herd size nor geographical location suggests a wide circulation over the country of the HEV, which is indeed a ubiquitous problem for both intensive and domestic farms. Further research is needed to identify farm-level factors that explain these results to establish future measures for HEV controls. The findings obtained in this study will also contribute to future risk assessment of HEV transmission through pork at slaughter level.

## Data availability statement

The datasets presented in this article are not readily available because they contain personal information. Requests to access the anonymised datasets should be directed to the corresponding author.

## Author contributions

Conceptualization and resources: ID, GA, and EP. Methodology: GI, RS, GLA, and LD. Validation: GI, RR, DD'A, and FO. Formal analysis: GI, LD, RS, DD'A, and FO. Data curation: FO and ID. Writing—original draft preparation: ID, GI, EP, and FO. Writing—review and editing: LD, GLA, RR, DD'A, GG, SS, FO, RS, and CM. Funding acquisition: ID, GA, GLA, EB, and EP. All authors have read and agreed to the published version of the manuscript.
